# The tenocyte phenotype of human primary tendon cells in vitro is reduced by glucocorticoids

**DOI:** 10.1186/s12891-016-1328-9

**Published:** 2016-11-10

**Authors:** Christoph Spang, Jialin Chen, Ludvig J. Backman

**Affiliations:** 1 Department of Integrative Medical Biology, Anatomy, Umeå University, SE-901 87 Umeå, Sweden; 2 Dr Alfen Orthopedic Spine Center, 97080 Würzburg, Germany

**Keywords:** Dexamethasone, Scleraxis, Collagen, Cell viability, Phenotype, Tendinopathy, Tenocytes, Tendon cells

## Abstract

**Background:**

The use of corticosteroids (e.g., dexamethasone) as treatment for tendinopathy has recently been questioned as higher risks for ruptures have been observed clinically. In vitro studies have reported that dexamethasone exposed tendon cells, tenocytes, show reduced cell viability and collagen production. Little is known about the effect of dexamethasone on the characteristics of tenocytes. Furthermore, there are uncertainties about the existence of apoptosis and if the reduction of collagen affects all collagen subtypes.

**Methods:**

We evaluated these aspects by exposing primary tendon cells to dexamethasone (Dex) in concentrations ranging from 1 to 1000 nM. Gene expression of the specific tenocyte markers scleraxis (*Scx*) and tenomodulin (*Tnmd*) and markers for other mesenchymal lineages, such as bone (*Alpl*, *Ocn*), cartilage (*Acan*, *Sox9*) and fat (*Cebpα*, *Pparg*) was measured via qPCR. Cell viability and proliferation was calculated using a MTS Assay. Cell death was measured by LDH assay and cleaved caspase-3 using Western Blot. Gene expression of collagen subtypes *Col1*, *Col3* and *Col14* was analyzed using qPCR.

**Results:**

Stimulation with Dex decreased cell viability and LDH levels. Dex also induced a significant reduction of *Scx* gene expression and a marked loss of fibroblast like cell shape. The mRNA for all examined collagen subtypes was found to be down-regulated. Among non-tendinous genes only *Pparg* was significantly increased, whereas *Acan*, *Alpl* and *Sox9* were reduced.

**Conclusions:**

These results indicate a Dex induced phenotype drift of the tenocytes by reducing scleraxis expression. Reduction of several collagen subtypes, but not cell death, seems to be a feature of Dex induced tissue degeneration.

## Background

Chronic tendon pain (tendinopathy) is a troublesome condition that often affects professional and recreational athletes but can also be found among non-active individuals [1–3]. The histopathological features (tendinosis) are mainly defined as structural degeneration characterized by reduced levels of type 1 collagen and haphazardly increased proliferation of smaller type 3 collagen and increased vascularization [4, 5]. Furthermore, cell proliferation and change in cell shape towards rounded and wavy cells, but also occasional cell death has been reported [4, 5]. In addition, it has been shown that in some areas of the tendon features of lipoid degeneration can occur [4]. Furthermore, adherence of fat rich peritendinous tissues onto the tendon proper has been observed [6, 7]. Calcification is another feature that is frequently present potentially being driven by an abnormal pathway of tendon healing [4, 8]. In areas with high levels of compressive loads fibrocartilaginous metaplasia has been observed [4, 9]. The mechanisms behind the tissue changes are not fully understood. However, there is some evidence that extensive load and the imbalance of molecules such as hormones and neuropeptides are involved [9–11].

Treatment methods for tendinopathies are often applied with low scientific evidence [12] and there are controversies about the use of some of them. Especially the application of glucocorticoids, e.g., the commonly used substance dexamethasone (Dex), is still heavily debated and questioned. Glucocorticoids in general are accepted as effective treatments for many inflammatory conditions [13]. For many years tendinopathies were considered as non-inflammatory and degenerative condition (“tendinosis”) by several authors as no signs of a classical inflammation was found in the tendon proper [14, 15]. However, recent studies using advances techniques have suggested that tendon degeneration may be an active process with involvement of aspects of inflammation-mediated responses especially in the early stages [16–18]. Clinical studies on the use of glucocorticoids for tendinopathies have reported short-term pain relief but poor long-term effects including a weakening of tendon structure on the longer perspective [19, 20]. In fact, many reports about spontaneous, often non-traumatic tendon ruptures following the use of these substances have been published [21–28].

In vitro studies have shown that exposure to glucocorticoids has negative effects on tendon cells [29]. It can lead to a reduction in cell viability, proliferation and reduced production of total collagen particularly collagen 1, and proteoglycans [30–35]. These effects are thought to weaken the tendon structure indicated by decreased mechanical properties [29, 36] and may eventually lead to higher risks for tendon ruptures. Conflicting data are available on whether apoptosis is a feature involved in this process [32, 37, 38]. Furthermore it is unclear if all collagen subtypes are affected by dexamethasone in the same way or if it is restricted to the collagen I subtype. As tendinopathy is indicated by an increased collagen 3 production [5] it is important to know how the expression of this subtype responds to glucocorticoids. The effect on collagen type 14, often found in areas of high mechanical stress [39, 40], needs to be evaluated as well.

Recently it has been reported that dexamethasone can influence the differentiation of tendon stem cells in vitro and that it thus can have an impact on the phenotype of cells towards non-tendinous like cells [41–43]. However, no attention has been paid on the potential effect on the expression of tendon specific markers in differentiated primary tendon cells, tenocytes. This is a drawback since this cell population makes up the vast majority of cells in the tendon. The primary purpose of this study was therefore to analyze the expression of tenocyte specific markers scleraxis and tenomodulin in response to Dexamethasone stimulation. Furthermore, the effect on the occurrence of cell death and the expression of collagen subtypes 1, 3 and 14 were evaluated.

## Methods

### Material and cell culture

Tendon cells used in the present study were derived from plantaris tendon samples from patients (*n* = 7; 5 men, 2 women; mean age 46 years) with chronic co-existing midportion Achilles and plantaris tendinopathy [44]. All patients had pain duration of at least 3 months and verified tendinopathies as shown by clinical examination including Ultrasound and Colour Doppler (US/CD). Exclusion criteria were acute and chronic inflammatory diseases, previous intratendinous treatments and partial tears in the Achilles tendon.

The tissue was obtained in the course of a previously described surgical procedure that includes plantaris tendon removal ﻿and Achilles tendon scraping [7, 45]. The isolation and culturing of primary tendon cells was carried out according to a protocol that has been routinely used in our laboratory [46]. Cells were cultured in DMEM supplemented with 10 % fetal bovine serum (FBS; Invitrogen, code: 16000), 1 % pen-strep (Invitrogen; code: 15140) and 0.2 % L-glutamine (Invitrogen; code: 25030) at 37 °C in a humidified atmosphere of 5 % CO_2_.

The cells in this study were used in passages 2–4. Prior to stimulation with dexamethasone cells were serum-starved for 24 h with 1 % FBS in DMEM. All experiments (see below) were performed at least three times and cells from at least two different patients were used.

### Ethical considerations

The principles expressed in the Declaration of Helsinki were followed. A written informed consent was received from all patients included in this study. Ethical approvals were obtained from the Ethical Committee at the Medical Faculty of Umeå University, and the Regional Ethical Review Board in Umeå (dnr 04-157 M; 2011-83-32 M).

### Immunocytochemistry

In order to characterize the primary tendon cells in vitro immunocytochemistry (ICC) was performed. Antibodies were directed towards hematopoietic progenitor cell antigen CD34 (1:25; Santa Cruz; code: sc9095), collagen 1 (COL1) (1:50; Abcam; code: ab34710), collagen 3 (COL3) (1:50; Abcam; code: ab7778), octamer-binding transcription factor 4 (OCT4) (1:50; Abcam; code: ab27985), scleraxis (SCX) 1:50; Abcam; code: ab58655), tenomodulin (TMD) 1:50; Abcam; code: ab203676).

For the procedure 15,000 cells/well were seeded on 8-well chamber slides overnight. The following day cells were initially fixed in 3.7 % paraformaldehyde in 1× PBS for 15 min. Then they were washed 4 times for 1 min in 1× PBS. After that cells were blocked with normal serum (1:20 in 1× PBS) for 15 min and incubated with the primary antibody for 60 min at 37 °C. Subsequently they were washed and blocked again, and incubated with the secondary antibody for 30 min at 37 °C. Eventually another washing step was performed before the slides were mounted using ProLong® Diamond Antifade Mountant with DAPI (Life Technologies; code: P36962). In order to evaluate the specificity of the immunofluorescence a control staining, where the primary antibody was replaced with 1× PBS, was performed. The immunoreactions were analyzed via a Zeiss Axioskop 2 plus microscope equipped with epifluorescence and an Olympus DP70 digital camera. The figure montages were created with Photoshop CS5 Extended software.

When using primary antibodies from rabbits, swine normal serum (1:20; Jackson I.R; code: 014-000-121) was used for blocking and swine anti-rabbit secondary antibody conjugated with tetramethylrhodamine isocyanate (TRITC) (1:40; DAKO; code: R0156) was applied. For goat antibodies, donkey normal serum (1:20; Jackson I.R; code: 017-000-121) and donkey anti goat secondary antibody conjugated with fluorescein isothiocyanate (FITC) (1:50; Jackson I.R.; code: 705-095-147) were used.

### Stimulation with dexamethasone (Dex)

Dexamethasone (Sigma; code: D4902) was solved in pure ethanol and diluted into concentrations of 1 mM, 10 mM, 100 mM and 1000 mM. For exposure the Dex solutions were applied 1:1000 in 1 % FBS in DMEM (2 μl Dex + 2 ml 1 % FBS in DMEM). Thus the final concentrations of Dex were 1 nM, 10 nM, 100 nM and 1000 nM. The durations of stimulation (time points) were 6 h, 12 h, 24 h, 48 h, 72 h, 96 h and 120 h. The control, unstimulated cells, were exposed to pure ethanol using the same volume (2 μl ethanol + 2 ml 1 % FBS in DMEM).

### Measuring cell death (LDH assay)

Cell death in response to the Dex exposure was measured by a lactate dehydrogenase (LDH) assay from Promega (code: G1780). At each time point supernatant was collected and then immediately stored at -20 °C. For the analysis 50 μl of supernatant was pipetted into a 96-well plate, then mixed with 50 μl reconstituted substrate mix and incubated for 30 min in a light protected environment under shaking. After that 50 μl of stop solution was applied and the absorbance was read at 490 nm using a plate reader. LDH was measured at all time points and all concentrations described above.

### Measuring cell viability (MTS assay)

The effect of dexamethasone on the proliferation and viability of tendon cells was measured using a MTS assay (CellTiter 96® Aqueous One Solution Cell Proliferation Assay; code: G3581). For this purpose tendon cells were seeded in a 96 well plate overnight at a density of 5,000/well. For analysis MTS reagent (20 μl per 100 μl media) was added and incubated for 4 h at 37 °C, 5 % CO_2_. The amount of formazan produced by cellular reduction of MTS, was measured with a micro-plate reader and the absorbance of 490 nm. MTS was measured at all time points and all concentrations described above.

### RNA isolation, reverse transcription, and qPCR

For analyzing gene expression qPCR was performed. Genes related to collagens (*Col1*, *Col3*, *Col14*) were analyzed. Furthermore markers that are tenocyte specific (*Scx*, *Tnmd*) and those characterizing non-tendinous cells (bone: *Alpl*, *Ocn*; cartilage: *Acan*, *Sox9*; fat: *Pparg*, *Cebpα*) were looked at. All applied probes and primers are listed in Tables 1 and 2.

**Table 1:** Probes used for qPCR (Taqman; Applied Biosystems)

Gene		Amplicon length	Code
*Actb*	β-actin	63	Hs01060665_g1
*Col1*	Collagen type 1	66	Hs00164004_m1
*Col3*	Collagen type 3	65	Hs00943809_m1
*Scx*	Scleraxis	63	Hs03054634_g1
*Acan*	Aggrecan	91	Hs00153936_m1
*Alpl*	Alkaline phosphatase	79	Hs01029144_m1
*Pparg*	Peroxisome proliferator-activated receptor γ	90	Hs01115513_m1

**Table 2:** Primers used for qPCR (SYBR Green)

Gene		Sequence
*Gapdh*	Glyceraldehyde 3-phosphate dehydrogenase	F: TGACGCTGGGGCTGGCATTG R: GGCTGGTGGTCCAGGGGTCT
*Col1*	Collagen type 1	F: CGATGGATTCCAGTTCGAGTAT R: CATCGACAGTGACGCTGTAGG
*Col14*	Collagen type 14	F: AAGGATTGCCCTCCGACTACAC R: CTGATGCGTTCATTGCCTTCTC
*Cebpa*	CCAAT/enhancer-binding protein alpha	F: GCGGCGACTTTGACTACC R: GCTTGGCTTCATCCTCCTC
*Ocn*	Osteocalcin	F: GACACCATGAGGACCCTCTC R: GCCTGGTAGTTGTTGTGAGC
*Scx*	Scleraxis	F: CGAGAACACCCAGCCCAAAC R: CTCCGAATCGCAGTCTTTCTGTC
*Sox9*	SRY-box 9	F: GGCGGAGGAAGTCGGTGAAGAA R: GCTCATGCCGGAGGAGGAGTGT
*Tnmd*	Tenomodulin	F: TGGGTGGTCCCTCAAGTGAAAGT R: CTCGACGGCAGTAAATACAACAATA

Initially, RNA was isolated using an extraction kit (Qiagen; code: 74106). The extracted RNA was then reverse transcribed into cDNA via a High Capacity cDNA Reverse Transcription kit (Applied Biosystems; code: 4368813). The settings for the conversion which was performed on a thermal cycler (Eppendorf Mastercycler EP Gradient S) were as follow: 10 min at 25 °C, 120 min at 37 °C, 5 min at 85 °C.

For the quantitative PCR (qPCR) either TaqMan fast universal PCR mastermix (Applied Biosystems; code: 4352042) or SYBR Green fast universal PCR mastermix (Applied Biosystems; code: 4385612) was used. For each of the amplifications, 40 ng of cDNA was taken and analyzed in technical dublicates.

The amplification was performed in a ViiA7 Real-Time PCR system (Applied Biosystems). The expression levels of genes were calculated in relation to the endogenous control β-actin (*Actb*) for the Taqman probes and *Gapdh* when using SYBR Green. For more practical details see [46, 47]. mRNA was analyzed after stimulation with 100 nM Dex after 12 h, 24 h, 48 h and 72 h. For *Scx* and *Tnmd* all concentrations (1nM, 10nM, 100nM, 1000nM) were used.

### Western blot

Cells were washed in sterile 1× PBS and then stored in -80 °C until all time point were collected. On the day of analyses cells were scraped in lysis buffer (RIPA) supplemented with protease inhibitor (1:200), put in an Eppendorf tube, vortexed and then incubated on ice for 30–60 min. After that the tubes were centrifuged to remove the cell debris. The supernatant was taken and analyzed for concentrations of total proteins using Protein Assay Dye Reagent Concentrate (Bio-Rad; code: 500–0006). Bovine Albumin Serum (BSA; Sigma; code: A9647) was used as a standard. Before loading onto a SDS-PAGE gel, in similar protein concentrations, the samples were boiled in 2× Lammeli buffer (Bio-Rad; code: 161–0737) supplemented with beta-mercaptoethanol. After the run (150 V) the proteins were transferred to a polyvinylidene fluoride transfer membrane for 1 h at 100 V. The membrane was then blocked with either 5 % BSA or 5 % non-fat milk powder in 1× TBS-T for 60 min and eventually incubated with the primary antibody at 4 °C overnight. Next day the membrane was washed in TBS-T (3×5 min) and after that incubated with the secondary antibody at room temperature for 60 min. After the final wash the membranes were treated with chemiluminescent HRP substrate (GE Healthcare; code: RPN2232) for 5 min and then visualized using Odyssey® Fc imaging system (LI-COR, Lincoln, NE, USA).

Antibodies were directed towards cleaved caspase 3 (cCasp3) (Cell Signaling; code: 9664), COL 1 (Cell Signaling; code: 9496), SCX (Abcam; code: ab58655) and TMND (Abcam; code: ab203676). Stimulation with Dex was performed in a concentration of 100 nM for 12 h, 24 h, 48 h and 72 h.

### Oil Red O staining

For the detection of adipocytes Oil Red O staining was performed. Before that cells were stimulated with Dex every second day for 21 days and media containing Dex (100 nM) was replaced every second day.

Cells were initially fixed in 3.7 % paraformaldehyde for 30–40 min. After that they were washed with 1× PBS (3x5 min) and rinsed in water twice. After that Oil Red O Solution was applied for 50 min. Cells were then washed with water and eventually stained with hematoxylin (10 min).

### Statistical analysis

Data were statistically analyzed by using PASW Statistics 18 (SPSS). One-way analysis of variance (ANOVA), followed by the Bonferroni post-hoc test, and independent t-tests were used. All results were successfully reproduced at least twice and performed in triplicates and technical duplicates. Significance levels were predetermined at *p* < 0.05.

## Results

### Primary tendon cells express tenocyte markers

**Fig. 1:**
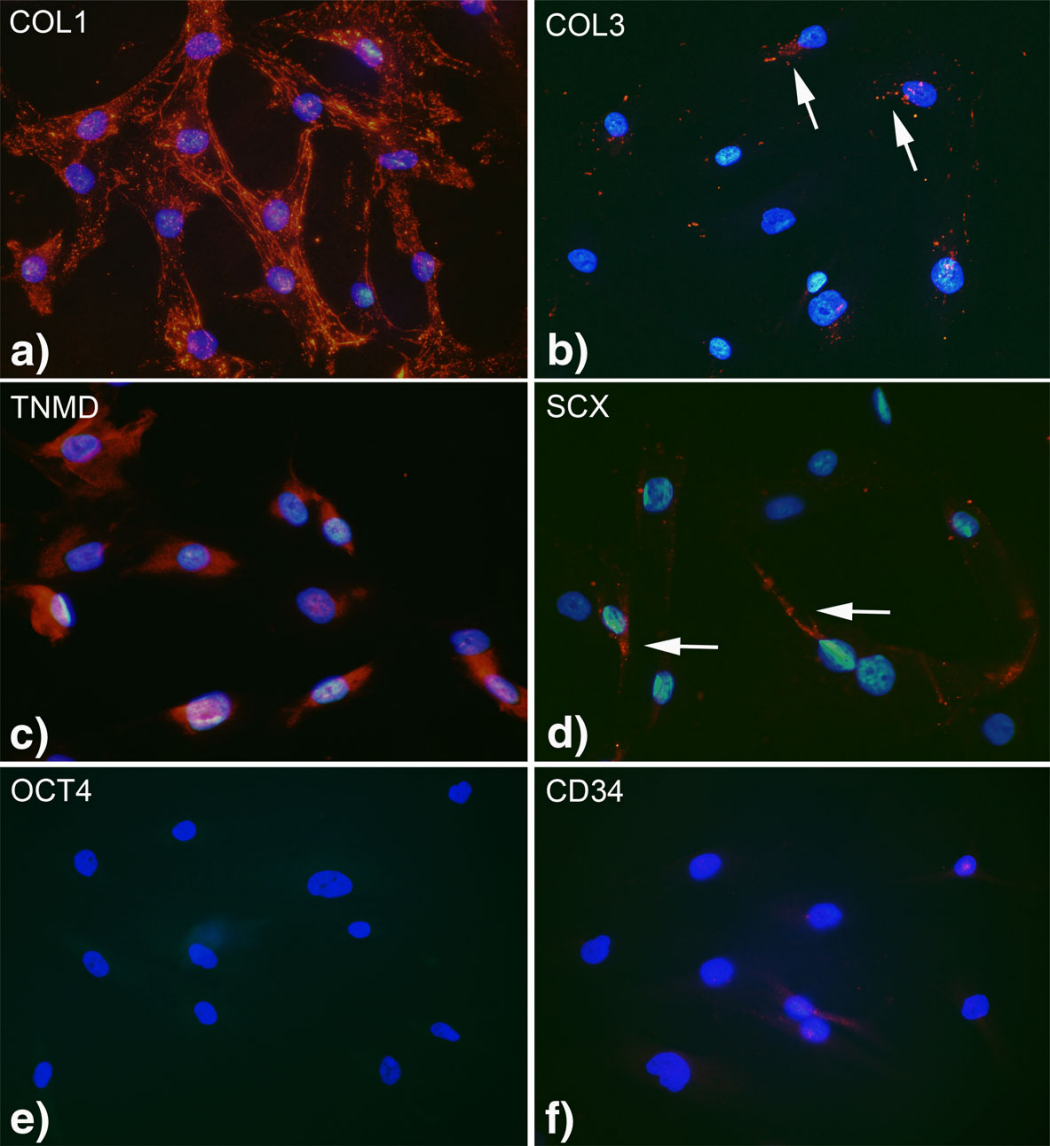
Primary tendon cells stained for COL 1 (**a**), COL 3 (**b**), TNMD (**c**), SCX (**d**), OCT4 (**e**) and CD34 (**f**). Immunoreactions are seen for COL1, COL3 (*arrows*), TNMD and SCX (*arrows*). No or very little reactions can be seen for OCT4 and CD34

The primary cells revealed immunoreactions for COL 1, SCX, TNMD and to some extent COL 3 (Fig. 1a-d). No, or very little, reactions were seen for stemness marker OCT4 and endothelial marker CD34 (Fig. 1e, f).

### Decrease of cell death, cell viability and proliferation

**Fig. 2:**
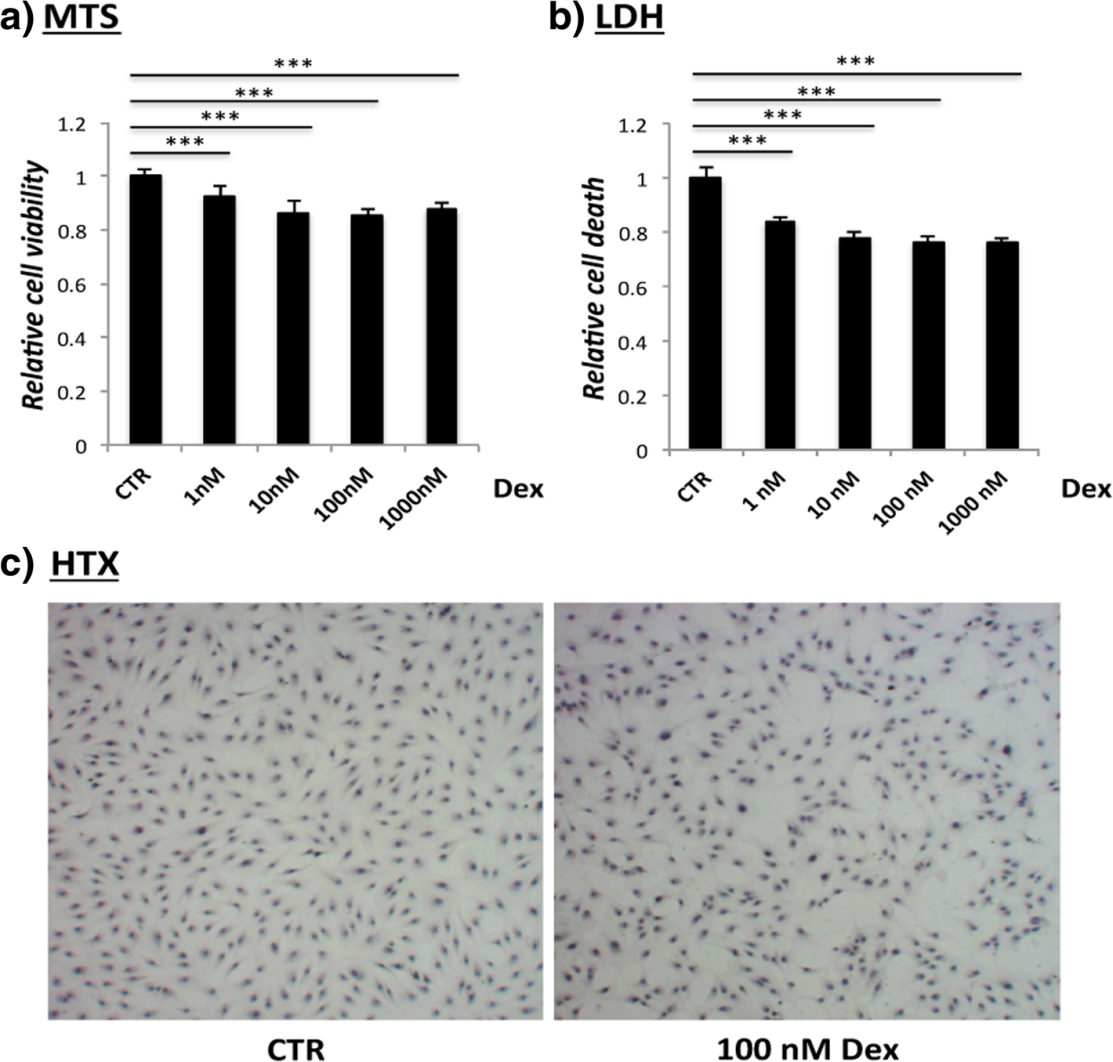
Effects of Dex on cell viability (*MTS*) and cell death (*LDH*) levels. A significant reduction in cell viability (**a**) and LDH (**b**) after 72 h of Dex treatment is found. Microscopic pictures of stimulated cells (7 days) stained with Oil Red solution and hematoxylin show lower cell numbers and a loss of fibroblastic appearance after treatment with Dex (**c**). *** *p* < 0.001

The exposure of Dex leads to a dose-depended reduction of cell viability after 72 h (Fig. 2a). Similar trends could be observed also at earlier time point (not shown).

The LDH assay showed a dose-depended reduction of cell death after 72 h (Fig. 2b) with similar trends for earlier time points. There was no presence of cleaved caspase-3 (not shown) after 24 h. The reduced number of cells is also seen macroscopically (Fig. 2c).

### Decreased *Scx* gene expression after Dex treatment

**Fig. 3:**
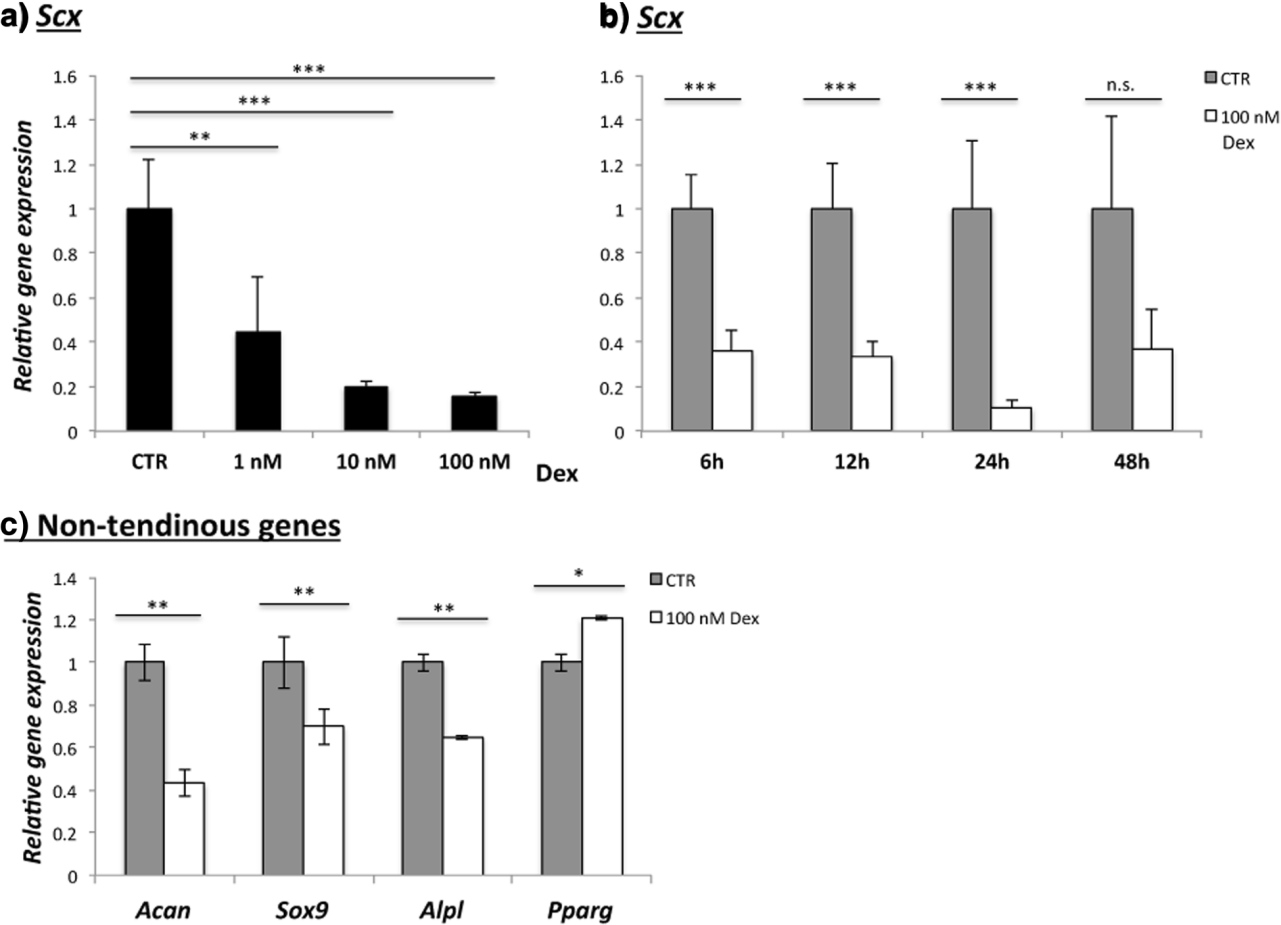
Results of Dex (100 nM) exposure on the relative *Scx* gene expression and the expression of non-tendinous genes. A significant down regulation of *Scx* mRNA is seen (**a**) and this down regulation is consistent over a 48 h time course (**b**). *Acan*, *Sox9* and *Alpl* expression is reduced (**c**) and *Pparg* is up-regulated in cells exposed to Dex. *p < 0.05; ** *p* < 0.01; *** *p* < 0.001; n.s. (non-significant)

The expression of scleraxis (*Scx*) mRNA was reduced (Fig. 3a, b) after Dex treatment at all time points examined, most prominently after 24 h. Gene expression for *Tnmd* was very low and not considered for analysis.

### Effects of Dex on tendon cell (tenocyte) phenotype

Among the analyzed non-tenocyte genes, only the fat marker *Pparg* was significantly up-regulated on mRNA level after Dex treatment (Fig. 3c). However, Oil red O staining after 21 days of stimulation did not show any presence of adipocytes in the culture (not shown).

*Acan*, *Sox9* and *Alpl* were found to be decreased (Fig. 3). The gene expression of *Ocn* and *Cebpα* was very low in both groups.

Stimulated cells exhibited a different shape than untreated cells. They lost their typical elongated fibroblastic appearance (Fig. 2c).

### Decreased expression of collagens

**Fig. 4:**
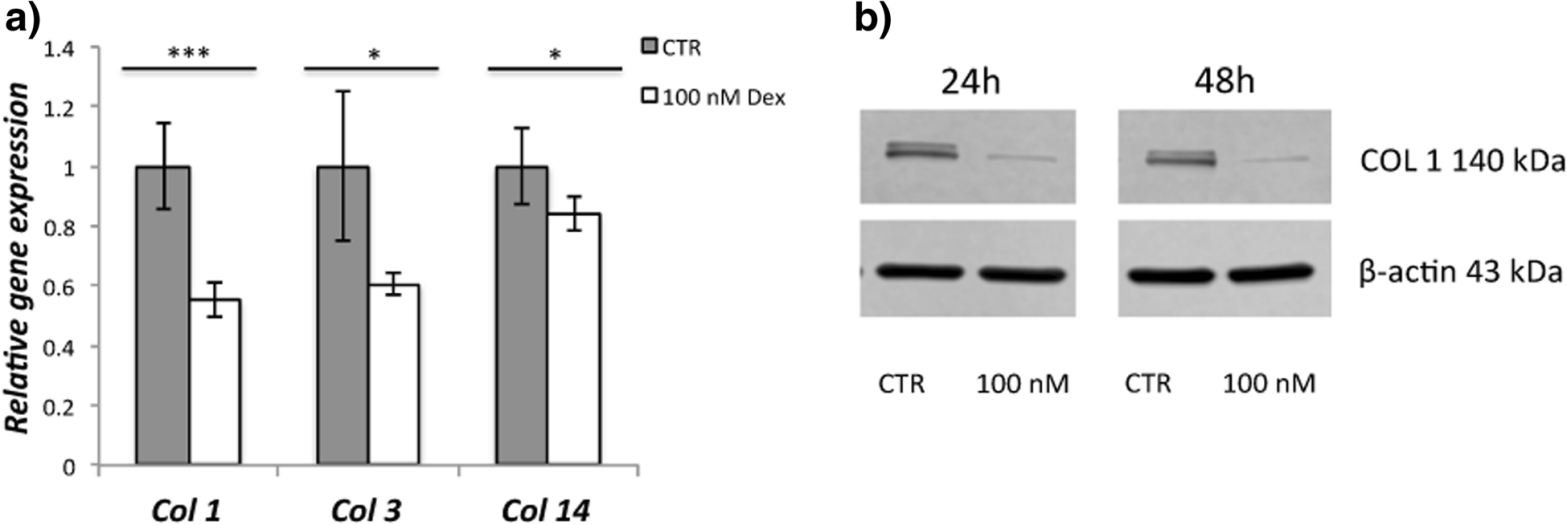
Results of Dex (100 nM) exposure on the expression of collagens. mRNA of collagen subtypes *Col 1*, *Col 3* and *Col 14* is significantly down-regulated after 24 h of Dex exposure (**a**) . COL 1 protein is decreased after 24 h and 48 h of Dex exposure (**b**). * *p* < 0.05; *** *p* < 0.001

Dex stimulation resulted in decreased expression of *Col1*, *Col3* and *Col14* on mRNA level after 24 h (Fig. 4a), and similar results for other examined time points (24 h, 48 h, 72 h, 96 h). COL 1 was found to be reduced on protein level as well (Fig. 4b).

## Discussion

This is to our knowledge the first study evaluating tenocyte marker expression in response to exposure of the corticosteroid Dexamethasone (Dex) in primary tendon cells. Our results suggest that Dex leads to a marked reduction of scleraxis, a transcription factor that has been reported to be essential for tendon development and differentiation [48]. At the same time we found that the tendon cells lost their typical fibroblastic shape during the stimulation. This indicates a potential phenotype change of the tenocytes as a result of Dex. When evaluating the expression of non-tendinous markers, we found that the adipocyte marker PPARG was up-regulated. However, staining for adipocytes did not show a presence of adipocytes within 21 days of stimulation. That is not very surprising since studies have shown that compared to tendon stem cells, primary cells have a very low potential to differentiated into cartilage-, fat- and bone related cells [49]. Studies on tendon stem cells have reported a phenotype change towards fat cells via reduction of scleraxis [41–43]. However, clone formation experiments prior to the present study have indicated that the cells in culture in the present study are mature tendon cells and that the amount of possible stem cells was very little. Thus, at least the marked reduction of scleraxis must be related to changes occurring in primary matured tendon cells. In fact, not all processes of tenocytes differentiation are fully understood yet. Therefore, the reduction of scleraxis accompanied with the change in cell shape in response to Dex could be a sign of reduced tenocyte phenotype, which may potentially affect the tendon. More studies are needed on how this scleraxis reduction affects the cell on a downstream direction.

Another observation of this study is a decrease in cell viability. This has been reported by other studies before [30–32]. Surprisingly, this reduction was not associated with an increase of cell death, as indicated by the reduction of LDH levels in our study. Furthermore, we did not find cleaved caspase-3 by Western Blot. This may indicate that apoptosis/necrosis is not a feature of Dex exposure as previously reported by Wong et al. on primary patellar tendon cells [32]. In fact, there are conflicting data in the literature [32, 37, 38]. These discrepancies might be explained by different concentrations of Dex and the use of cells from different tendons in these studies. In our study, we did not find any signs of increased cell death processes in cells derived from plantaris tendinopathy tendons, with different concentrations (1 nM-1000 nM) of Dex treatment within 5 days. However, it should also be mentioned that LDH does not necessarily detect apoptosis. Necrotic processes are even more likely to be detected since it measures plasma permeability. Based on our results, it rather seems that the turnover in general, i.e., both proliferation and cell death (necrosis/apoptosis), is lower in stimulated cells as both cell viability and cell death is decreased. That doesn’t mean that there is a general decrease in cell death as these results are not normalized. In fact, Poulsen and co-workers have suggested that glucocorticoids can cause senescence in tendon cells, which can explain the reduced cell viability [50].

It is also known that patients with Cushing syndrome (CS) resulting from chronic exposure to excessive circulating levels of glucocorticoids have major alterations in the tendon structure and/or tendon function [51]. Despite based on only a few studies, it shows that chronic use of glucocorticoids harms the tendon even if used systemically.

The third finding of the present work was that Dex not only reduced the expression of collagen 1 but also mRNA of collagen 3 and 14. Previous studies have shown that collagen 1, the main collagen in tendons, is reduced after exposure to corticosteroids [30]. Collagen 3, a smaller collagen, is another collagen type in tendons and known to be up-regulated in the process of tendinopathy [5]. Also collagen 14 is present in tendons [39]. Type 14 collagen is often found in areas of high mechanical stress [40]. The reduction of all examined collagen subtypes indicates that Dex may lead to a weakening of tendon structure. It is therefore not surprising that spontaneous ruptures have been linked to injections of corticosteroids in tendons [21–28]. Our results give a plausible molecular explanation for that.

A drawback of this study is that it is unclear if the Dex concentrations used in vitro are representative for the in vivo situation in the tendon when it is clinically used. However, we applied a wide range of concentrations and stack to those used in previous studies. Another issue is that most mRNA and protein analyses were solely performed after 100nM Dex exposure. The effect of other concentrations was only measured for MTS and LDH. We had tested other concentrations prior to these experiments. Based on the results of these and the fact that 100nM Dex had the highest impact on cell viability we decided to use it for further experiments.

## Conclusion

Overall, the results of our study suggest that Dex reduces the tenocyte phenotype via reduction of Scx expression, decreased cell turnover and decreased expression of collagen 1, 3, 14 in primary tendon cells. This study suggests that Dex may weaken the tendon structure in vivo.

## Abbreviations

Acan: Aggrecan
Actb: β-actin
Alpl: Alkaline phosphatase
Cebpa: CCAAT/enhancer-binding protein alpha
Col1: Collagen type 1
Col14: Collagen type 14
Col3: Collagen type 3
CS: Christoph Spang
Dex: Dexamethasone
Gapdh: Glyceraldehyde 3-phosphate dehydrogenase
JC: Jialin Chen
LDH: Lactate dehydrogenase
LJB: Ludvig J Backman
Ocn: Osteocalcin
Pparg: Peroxisome proliferator-activated receptor γ
Scx: Scleraxis
Sox9: SRY-box 9
Tnmd: Tenomodulin
